# Supplementary material for: Are carbon emissions trading and green financial instruments synergistic? -Comprehensive quantitative research based on content analysis
Source: PLoS One. 2024 Mar 7;19(3):e0298601. doi: 10.1371/journal.pone.0298601 (PMC10919601; doi:10.1371/journal.pone.0298601)
Supplement: S1 Data — (ZIP) [file pone.0298601.s001.zip › Data/地级市Z_F工作报告.zip!/║■▒▒/╦μ╓▌╩╨/└┤╫╘╣1⁄2╓┌║┼í╛┬φ┐╦╩2╛▌═°í┐_2013.docx]

各位代表：

现在，我代表市人民政府向大会报告政府工作，请予审议，并请市政协委员和各位列席人员提出意见。

一、过去一年工作回顾

刚刚过去的2012年，是随州发展史上极不平凡的一年。一年来，我们在省委省政府和市委的正确领导下，深入贯彻科学发展观，坚决执行“五进”方针，全力推进“圣地车都”建设，扎实开展“四大行动”，紧紧团结依靠全市人民，沉着果敢应对经济下行压力，艰苦卓绝抗击罕见自然灾害，砥砺前行，奋勇争先，较好完成了市三届人大一次会议确定的各项任务，逆势而进开创了科学发展新局面。

——战略定位跃升新高度。“圣地车都”建设上升为省级战略，随州的发展定位更加明确。我市与20多个省直部门签署了共建“圣地车都”协议，加强多方合作，争取更大战略支持。区域发展更加协调：随县成功创建省旅游强县，延期享受省直部门对口帮扶政策；广水市被纳入大别山革命老区经济社会发展试验区，被授予中国风机名城；曾都区成为省级服务业综合改革试点，被授予省科技创新先进区。

——综合实力迈上新台阶。主要经济指标增幅均高于全省全国平均水平。预计实现地区生产总值580亿元，较上年增长12%，经济总量占全省的比重提高0.2个百分点，三次产业比例调整为19：48：33，县域经济和开发区经济实力有新提升。完成规模工业增加值230亿元，同比增长17%。全社会固定资产投资达500亿元，同比增长30%，增幅全省靠前。地方一般预算收入22.99亿元，同比增长34.7%。农产品出口总额保持全省第一，利用外资增幅居全省前列。

——城市形象展现新风貌。中心城区快速发展，规划工作得到加强，年初确定的25个城市功能项目全面实施。“三城同创”持续推进，创建全国绿化模范城市通过考评验收，省城市综合管理排名继续靠前。广水和随县县城建设加快，大洪山建设开发进展显著。继应山办事处、广水办事处之后，北郊、东城办事处进入全省百强乡镇，新增19个宜居村庄。世界华人炎帝故里寻根节继续成功举办，全省县域经济工作会议在我市成功召开，城市知名度美誉度不断上升。

——人民生活实现新改善。城镇居民人均可支配收入19164元，农民人均纯收入8466元，同比分别增长13%和14%。物价水平总体稳定，居民消费价格涨幅为3.2%。市政府年初承诺的十件实事全面或超额完成，一批事关群众生活的问题得到较好解决。

——政府效能得到新提升。进一步强化效率观念和服务意识，政府执行力不断提高。认真落实市人大及其常委会的决议决定，充分听取政协意见，办理建议提案243件，满意率和基本满意率达到99%。深入开展 “作风整顿大行动”，查处“不在状态”党员干部130多人。大力加强勤政廉政建设，突出抓好工程建设等领域专项治理。实行电视问政，积极整改纠偏。新一轮行政审批制度改革正式启动。

一年来，我们积极应对复杂形势，各项工作都有新进展。

（一）抗旱救灾取得胜利，农业经济总体平稳。面对60年不遇的特大干旱，及时开展“生产自救、灾后帮扶大行动”，紧急组织抗旱工作队进村入户，市财政直接支出抗灾资金2300万元，多方累计投入近3亿元，最大限度降低灾害损失，确保了农业形势总体稳定。夏粮同比增长6.6%，全年粮食总产154.3万吨。特色农业面积突破40万亩，蔬菜、果茶、畜牧、水产全面增产，实现农产品加工产值340亿元，同比增长25%。加快农田水利建设，实施土地整治23万亩，完成106座小型水库的除险加固，整修堰塘2.1万口，打井1万多口，农村安全饮水普及率达到74%。新建通村水泥路299公里。全面实行封山育林，森林覆盖率达到50.45%。

　　（二）千方百计帮扶企业，工业平稳较快发展。果断出台应对经济下行的十条措施，深入开展“一企一策、对口帮扶”，与企业同舟共济。规模工业加快壮大，实现总产值855亿元，同比增长25%。中国专用汽车之都建设迈出坚实步伐，我市被认定为国家专用汽车高新技术产业化基地，汽车机械产业实现产值270亿元，同比增长13%。建材工业产值首次突破百亿元；化工、医药、食品加工业快速发展，产值增幅均超过30%。齐星集团、同星农业、程力集团、茂盛生物、华鑫冶金、三友食品6家企业入围“2012湖北民企百强”。科技创新进一步增强，高新技术企业达到36家，高新技术产业增加值占GDP的比重达到8.45%，新增2个中国驰名商标。市场主体快速成长，净增规模企业82家，新登记市场主体超过2万户。县域工业亮点纷呈，开发区和工业园区平台环境不断改善，69个投资过亿元的项目相继开工。

（三）第三产业保持繁荣，内需活力较大增强。第三产业加快发展，占GDP的比重达到33%，较上年有所提高。重点景区建设步伐加快，大洪山慈恩寺、三潭景区提档升级工程全面推进，淮河源西游记漂流正式营运，文化旅游产业增加值进一步提升。全年接待游客1210万人次，旅游综合收入74亿元，增幅均超过40%。内需拉动有所增强，社会消费品零售总额273亿元，同比增长16%。建筑和房地产业有序发展。充分发挥工商、质监、食品药品监督的职能作用，农资、成品油、酒类、烟草等专业市场秩序不断规范，消费环境进一步改善。

（四）项目建设强力推进，改革开放不断深入。扎实开展“项目建设大行动”，续建、开工500万元以上项目1390个。投资结构不断优化，预计完成工业投资277亿元，同比增长53.6%，居全省前列。厦工楚胜专汽、三环铸造二期、炎帝科技、广彩印刷等项目进展顺利，封江水源供水复线工程全面开工，麻竹高速随州西段、大洪山内循环公路、广水城区应急供水工程等项目启动实施。电网投资建设加强，新能源建设取得历史性突破，二妹山风电一期并网发电，武胜关风电场启动建设。要素保障有力，成功进入全省各类土地整治利用试点，耕地总量连续11年保持动态平衡，争取各类用地指标为历年最高。加大金融工作力度，全市当年净增贷款51亿元；成功发行7亿元城投债券，开创了我市直接融资先河。招商引资取得新成效，实际利用内联资金110亿元，同比增长37%；实际利用外资7200万美元，同比增长16%。改革开放取得新进展，营业税改增值税完成阶段性任务，事业单位分类改革有序推进，农村商业银行挂牌营运。外经外贸难中求进，食用菌出口2.2亿美元，继续领先全国地级市；机电产品出口总量首次超过农产品，出口结构得以优化。

（五）加大民生保障力度，社会事业协调发展。财政支出向民生倾斜，占总支出75%。落实就业政策，城镇新增就业1.7万人，下岗失业人员再就业3280人，农村劳动力转移就业1.8万人。五项社会保险参保突破百万人次，城乡居民养老保险制度全覆盖，上调企业退休人员养老金，为80岁以上老人发放高龄补贴。提高城乡低保待遇，社会救助惠及13.6万人，农村贫困人口减少1万人。住房公积金制度扩面1.5万人，保障性安居工程惠及10192户，其中1747户城镇困难家庭搬进新居。教育办学行为逐步规范，各县市区均被纳入省义务教育均衡发展实验县。加大文化惠民力度，建成50处农民体育健身工程和一批社区文化活动场所，完成1430个广播电视“村村通”工程建设。叶家山西周古墓群、文峰社区春秋墓地考古发掘产生深远影响。卫生应急和疾病预防管理工作走在全省前列，三年医改目标基本实现。计划生育责任目标全面完成，出生人口性别比逐步趋于正常。城市综合交通秩序整治初见成效，创建4条市容环境美好示范路，城市人均公共绿地面积9.38平方米；饮用水质总体达标，空气环境质量达标率90.4%，环保“五项约束性指标”全面完成考核任务。

与此同时，新闻出版、文联、外事侨务、对台、对口援疆工作不断加强，民族宗教、妇女儿童、红十字会、无偿献血、慈善、老龄和残疾人事业持续发展，审计、统计、国调、司法、档案、保密、地方志和烟草专卖等工作均取得新成绩。

（六）精神文明建设、民主法治建设进一步加强。广泛开展精神文明创建活动，加强思想道德建设，“湖北好人”左光满和“抗旱支书”张小林先进事迹影响广泛。开展法治随州创建活动，加强和创新社会管理，城市社区网格化管理有序推进，社区“三自”物业、农村 “五自”和村务协理员管理服务模式得到推广。深入开展 “平安建设大行动”，各类案件发案率继续下降，破案率上升，公众安全感增强。加强信访维稳工作，调解矛盾纠纷近万件，化解各类积案、难案200多件。安全生产态势总体平稳。进一步加强国防动员、民兵预备役和双拥共建工作，驻随部队、消防官兵应对急难险重任务的能力明显提升，工会、共青团、妇联等群团组织作用得到充分发挥，气象、水文、人民防空、供水供电、移动通信、无线电管理等工作保障有力。

各位代表！过去一年，我们取得了弥足珍贵的辉煌成绩。这些成绩，是全市上下贯彻执行中央和省委省政府各项方针政策的结果，是市委坚强领导及市人大、市政协加强监督支持的结果，饱含了各位代表、各位委员的智慧和心血，凝聚着全市人民的辛勤和汗水。在此，我代表市人民政府，向全市人民，向所有纳税人，向各位人大代表、政协委员，向各民主党派、工商联、人民团体和无党派人士，向驻随部队、武警官兵和政法公安干警，向关心支持随州发展的老领导和各界朋友们，表示衷心的感谢并致以崇高的敬意！

在总结成绩的同时，我们也清醒看到，全市经济社会发展中还存在不少困难和问题：一是经济总量不大，产业结构不优，工业化水平、城镇化率低于全省平均水平，面临赶超及转型的双重艰巨任务。二是严重旱灾影响尚未完全消除，农业生产和农民增收压力较大；受宏观经济形势的影响，工业增速明显放缓，要素制约仍较突出。三是财政实力有限，收支矛盾突出，保障能力亟待提高。四是有些部门和部分公务人员作风不实、执行不力，发展环境仍需优化等。对此，我们一定高度重视，努力加以解决。

二、2013年主要工作安排

2013年，是全面贯彻落实党的十八大精神的开局之年，是实施“十二五”规划的攻坚之年，是为全面建成“小康随州”奠定坚实基础的关键之年。当前，尽管世界经济进入深度转型调整期，我国经济进入中速增长期，经济下行压力依然较大，区域竞争更趋激烈，我市发展面临不少困难。但宏观经济走势总体向好，产业梯度转移加快，特别是党的十八大和省第十次党代会的胜利召开，开启了全面建成小康社会和“五个湖北”建设“黄金十年”新周期，为我们提供了难得的窗口机遇期。面对新的形势和挑战，要更加自信、自醒、自强，争取在新一轮发展中赢得主动！

围绕“圣地车都”战略实施，要立足2013年，积极谋划好未来三至五年的工作，确保“十二五”规划顺利实施，确保与全省同步建成小康社会。编制完成“圣地车都”战略规划，构建战略实施体系，细化落实“五个湖北”建设的措施，切实加强动态战略管理。将已出台的三年加速发展计划纲要作为执行“圣地车都”战略的核心抓手，力争实现固定资产投资、财政收入、工业增加值等主要投入产出指标三年倍增，加快打造“五地一枢纽”，加快向千亿元地区生产总值迈进。一是实施工业三年倍增计划。突破性发展汽车机械、农产品加工“两大龙头”，壮大冶金建材、医药化工、电子信息、战略性新兴产业“四个支柱”，力争2015年规模工业增加值突破500亿元。二是实施“神韵随州”城镇化加速计划。坚持走彰显文化、产业、生态特色的新型城镇化道路，中心城区向着“双百”大城市迈进，到2015年基本完成百平方公里框架建设，全市城镇化率尽快缩小与全省平均水平的差距。三是实施文化旅游产业支柱化培育计划。擦亮城市文化名片，大力开发旅游资源，力争到2015年文化旅游产业增加值占GDP的比重达到10%。四是实施基础设施保障支撑计划。加快基础设施项目投资建设，完善综合交通物流体系、能源电力供应保障体系、国土资源可持续利用体系、水资源及生态安全保障体系、多层次金融市场体系和信息化多领域利用体系。五是实施幸福民生普惠计划。以促进收入增长和实现充分就业为主线，充分发扬民主，切实改善民生，广泛凝聚民心，切实提升人民群众幸福指数。

实现2013年良好开局至关重要。今年政府工作的指导思想是：全面贯彻落实党的十八大和中央、全省经济工作会议精神，以科学发展观为指导，紧紧围绕“五个湖北”建设和“圣地车都”战略，牢牢把握“竞进提质”这一总要求，以“四化”同步为路径，以新一轮“四大行动”为抓手，以项目和投资为支撑，以改革创新为动力，加快提升工业竞争力、农业综合生产能力、城镇承载力、文化旅游影响力，不断壮大开发区经济、县域经济和开放型经济，着力赶超转型，建设“神韵随州”，增进民生福祉，稳神竞进，好中求快，扎实开局，全力推动经济社会科学发展、跨越式发展。

全年经济社会发展的主要预期目标是：地区生产总值增长12%左右，规模工业增加值增长17%，全社会固定资产投资增长26%以上，地方一般预算收入增长15%，外贸出口增长11%，实现内联资金、实际利用外资分别增长20%和10%，社会消费品零售总额增长16%，城镇居民人均可支配收入增长11%以上，农民人均纯收入增长12%，居民消费价格涨幅控制在4%左右，人口自然增长率控制在6.5‰以内，单位生产总值能耗下降3%，全面完成节能减排等各项考核目标。

上述目标是立足于现实基础与战略要求，着眼于需要与可能而提出来的，是必须确保完成的“底线”标准。实际工作中，我们将自我加压，按照三年加速发展的要求，确定在全省“保五争三”的奋斗目标，力争更好的结果。

新的一年，将重点抓好以下九个方面的工作。

（一）着力扩大经济规模总量，提质增效发展。牢牢把握发展实体经济这一坚实基础，发挥和提升第一产业的传统优势，突破性推动第二产业扩量提质增效，切实提升第三产业的支撑服务能力，推动特色发展，加快转型升级，促进经济步入新一轮上升通道。

狠抓投资、消费、出口，发挥引擎功能。坚持把扩大投资作为推动经济增长的主动力，进一步增加投资总量，不断优化投资结构，重点投向先进制造业、现代服务业、重大基础设施、社会发展等领域，确保今年完成全社会固定资产投资630亿元，其中工业投资占比达到50%以上。坚持把扩大内需作为战略基点，以主城区、县域商业中心和建制镇为重点，完善城乡流通体系和便民消费服务设施，扶持商贸流通企业，加快平价商店建设，积极培育消费热点，力争实现社会消费品零售总额320亿元。加强市场监管，保障消费安全。坚持把外经外贸作为对外开放的着力点，加快企业“走出去”的步伐，开拓国际市场，扩大机电产品、巩固农副产品、推动高新技术产品出口，力争出口总量达到8亿美元。

狠抓县域经济和开发区经济。坚持“四化”协调，推动县域经济均衡发展、争先进位。遵循开发区经济规律，着力夯实和拓展发展平台。市开发区围绕建设国家级开发区目标，进一步创新体制机制，优化服务环境，着力巩固提升城东、深圳工业园，尽快形成“一区多园”发展格局，主要经济指标增幅要高于全市平均水平、高于全省同类开发区平均水平。曾都开发区按照50平方公里、随县和广水开发区按照30平方公里规划，优化空间、功能和产业布局，提升聚集化发展水平。因地制宜发展一批定位明确、特色鲜明、设施配套、节约集约的乡镇工业园，打造一批经济重镇。

（二）着力推进新型工业化，做强做优做大。深入推进“工业兴市”战略，建立健全工业产业专业化推进的工作机制，加强工业经济运行监测分析和调度服务，确保工业三年倍增计划顺利实施，力争今年工业总产值达到1200亿元。

突出龙头支柱产业做强做大。按照产业集聚发展规律推动优势产业发展。进一步提升中国专用汽车之都竞争力，巩固部省共建、厅市会商成果，加快构筑专用汽车信息、检测、会展、营销、物流“五大中心”平台建设，加强资源整合、市场整顿，引导企业强强联合、细分市场，大力支持整车生产及关键零部件企业技改扩能。实施精品名牌战略，狠抓质量管理，开发高端产品，提高市场占有率。重点支持齐星集团、厦工楚胜等企业壮大整车规模，支持随州车轮、全力机械等企业做大做优汽车零部件产业。进一步推进“中国风机名城”建设，抢抓国家推广风机、水泵等节能产品的政策机遇，鼓励企业定向开发新产品，提升风机制造业在国内的行业地位。突破性发展农产品加工业，继续实施“亿只鸡”、“四个一批”工程，依托同星农业、三友食品、裕国菇业、炎帝科技、大自然农业等企业，扩大产业规模，提升加工转化能力，力争今年汽车机械、农产品加工产值双双突破350亿元。大力支持华鑫冶炼、 星化工、健民药业、波导电子等企业做大做强，推动冶金建材、医药化工、电子信息产业延伸链条，开发新型电子产品。加快发展新能源、新材料和节能环保等战略性新兴产业，培植新的增长点。严格矿产资源开发准入，在规范中健康发展石材等产业。

突出市场主体培育壮大。开展企业减负专项行动，深化“一企一策、对口帮扶”，完善“直通车”制度，实行无障碍、零距离、保姆式服务，着力解决企业面临的现实问题。加强分类指导，倾力支持龙头企业、上市后备企业和重点纳税企业。抓好中小企业成长工程，力争今年新增规模企业100家。落实推进全民创业的政策措施，确保新增市场主体稳中有升，新增小微企业1500家以上。

突出科技创新驱动。加大科技投入，提高自主创新能力和全民科技素质，加快建设创新型城市，力争新增高新技术企业10家以上。健全科技创新体系，加强产学研合作，支持组建工程技术研究中心、院士专家服务站、校企共建研发中心，推进省专用汽车研究院建设，积极创建省级科技孵化器和可持续发展实验区。鼓励企业加强技术创新、产品创新、品牌创新、产业组织创新、商业模式创新。

（三）着力加快农业现代化步伐，强农惠农富农。坚持以农业增效、农民增收为核心，推动农业农村经济转型发展。转变农业增长方式。按照“高产、优质、高效、生态、安全”的要求，加快推进产粮强市建设，调整粮食产品结构，扩大玉米、马铃薯等节水高效粮食作物面积，确保今年增产2.5万吨。大力发展特色农业，重点推进食用菌规模化、标准化种植，提高农产品出口在全国的领先地位。加快发展现代水产业、畜禽养殖业，抓好“菜篮子”工程。大力发展绿色生产，加强“三品一标”认证工作，确保农产品质量安全。探索发展休闲农业、旅游农业和非农产业，拓宽农民增收渠道。

推进农业产业化经营。突出区域特色、品质特色、功能特色，扩大农产品种养基地规模。统筹品种繁育、精深加工等重点环节，推动农业科技增收。大力支持龙头企业发展，进一步完善利益联结机制，力争今年新增省级龙头企业2家以上，新发展农民专业合作社100家以上，提高农民组织化程度。扶持发展农产品加工产业园，健全粮食产业体系和市场体系，推动市开发区及各县市区农产品加工园区提档升级，力争农产品加工产值达农业总产值的2倍以上。

改善农村生产生活条件。加快中低产田改造，继续实施土地连片整治。突出抓好防汛抗旱，加快实施中小河流治理，除险加固123座小型病险水库，高标准改造灌区干渠，健全完善小型水利设施管护机制，恢复和改善灌溉面积10万亩。加快实施“村村通”工程，建成通村公路400公里，完成900个村的广播电视工程建设任务。加快推进曾都区城乡一体化发展，在全市启动创建14个综合发展示范镇、20个新农村建设示范村，继续实施整村推进扶贫开发。

全面落实农村各项政策。积极解决二轮延包的历史遗留问题，坚持自愿、有偿的原则，流转土地35万亩，发展适度规模经营。坚持一村一策，引导农村集体经济发展，增强造血功能。加快转移农村富余劳动力，增加农民工资性收入。严格农民负担监管，绝不允许任何单位和个人以任何形式截留、克扣、挪用惠农资金。

（四）着力加速城镇化进程，建设“神韵随州”。“神在铸文化之魂，韵在绘山水之美”。坚持高起点规划、大手笔建设、精细化管理，突出深厚文化底蕴，提升城市能级和品牌，统筹城乡发展，力争今年城镇化率提高2-3个百分点。

构建充满活力的新型城乡统筹体系。努力把随州建设成为功能完善、特色鲜明、宜居宜业宜游的襄十随城市群核心城市、鄂豫门户节点城市。构建以中心城区为核心，以广水、随县城区为两翼，以重点镇为纽带，以农村新社区示范点为辐射的“一主两翼、多点支撑”城镇化统筹格局。中心城区尽快拉开百平方公里框架，做好山水、文化、产业文章，进一步明晰功能分区定位，实现新老城区融合、城市和文化融合。积极推进户籍制度改革，有序推进农村转移人口市民化。

提升规划管理科学化水平。编制城乡统筹与城镇化发展战略规划，启动城市总体规划修编，完善历史文化名城保护修建规划，制定城乡规划实施办法。健全规划委员会工作机制，引导公众参与规划、监督实施。加大规划执法力度，加强百平方公里规划区的分类管控，对建设项目全过程全方位监管，坚决遏制违法违规建设行为。

加快中心城区建设步伐。主要实施 “十大工程”：一是城南新区建设工程。重点启动“中国编钟音乐之都”核心区建设。二是316国道十岗至厉山段外迁工程。三是道路桥梁工程。建成青年路大桥，延伸白云大道至新白云湖大坝、编钟大道至麻竹高速连接线、炎帝大道至淅河十岗麻竹高速出口，改造青年路、烈山大道等主干道，提档升级20条小街小巷，进一步改善老城区基础设施。四是园林绿化工程。推进文化公园、回龙寺公园建设，启动白云山森林公园建设，延伸环白云湖滨湖风光带，进一步提高迎宾大道、炎帝大道绿化水平。五是节能减排工程。加快城南污水处理厂及配套管网建设，启动东护城河治理。六是民生保障工程。建设1100套公共租赁住房，改造1000户城市棚户区。七是市殡仪馆新建工程。八是商贸设施工程。加快碧桂园商业街建设，启动城市商贸综合体项目。九是公共服务设施工程。加快城市规划展览馆、滨湖体育运动场及有关大型公共文化医疗设施建设。十是城乡结合部整治工程。实施316国道明珠路以北及306省道南郊段环境整治。

巩固城市管理品牌。围绕构建“大城管”格局，加强协调联动，严格精细管理，确保城市综合管理工作在全省继续领先。进一步完善市政设施维护机制，规范市政设施命名，加强户外广告管理，推进城市亮化工程。严格市场经营秩序、交通秩序和停车秩序整治，着力解决交通拥堵问题。加强建筑渣土运输管理，切实解决鞭炮扰民、丧事扰民、噪音扰民和油烟扰民“四大顽症”，还市民一个安宁的居住环境。

（五）着力做强文化旅游产业，彰显“圣地”品牌。高质量编制文化旅游产业发展规划，创新体制机制，争取打造更高的产业发展政策平台，力争今年接待游客1450万人次，旅游综合收入90亿元，文化旅游产业增加值达到15亿元。

进一步推进世界华人谒祖圣地建设。强化经济性、商贸性和群众性，探索市场机制，高标准举办癸巳年寻根节，着力打造一流文化节庆品牌。积极创建海峡两岸交流基地和国家级爱国主义教育基地，切实增强全球华人的认可度和向往度，建好中华民族共有的精神家园。加快编制炎帝故里景区总体规划，启动三期建设，加快实施世界华人大宗祠等项目，完善配套设施。

进一步加强地域文化的保护、传承和利用。加强炎帝文化、编钟文化、曾随历史文化的研究和利用，加大非物质文化遗产的保护力度，深挖地域文化内涵。完成叶家山墓地二期考古发掘，争取随州大遗址片区纳入国家文化遗产保护项目，新建曾侯乙墓遗址展示厅，扩建市博物馆，保护和恢复古城风貌。谋划推进炎帝文化、西游神话等文化旅游产业园区建设，加快建设编钟文化软硬件载体，大力推进“中国编钟音乐之都”建设。

进一步加强重点景区创建工作。加快推进大洪山整体开发，基本建成慈恩寺景区，抓好游客服务中心、内循环公路等重点项目建设。进一步加大擂鼓墩、徐家河、三潭、中华山、洛阳千年银杏谷、桐柏山太白顶等景区的开发力度。大力推动炎帝故里创5A、4个景区创4A，积极创建旅游名镇。

进一步提升随州旅游整体影响力。大力开发特色文化旅游产品，推出精品旅游线路。优化整合资源，建立政府引导、企业主体、部门联动、区域协作的一体化营销模式，巩固周边市场，拓展国内及境外市场，不断提升随州文化旅游的市场知名度和产业影响力。

（六）着力提升区域承载服务能力，争创竞争优势。按照产城融合、产融融合的思路，降低综合商务成本，提高产业服务便利性可及性，增强区域竞争的发展承载力、资源聚集力和要素吸引力，促进人流、物流、信息流、资金流等要素汇集随州。

大力强化基础服务功能。坚持生产性服务业与生活性服务业并重，加快发展商贸购物、医疗保健、文化体育教育、社区服务，有序发展建筑和房地产业，积极培育引进专业咨询公司、法律服务、电子商务等中介机构，完善配套水、电、气等市政服务。

大力加快物流业发展。充分发挥我市地理区位与交通优势，结合产业特点科学定位物流业发展目标，制定规划和政策措施，加快建设区域性物流枢纽，积极推进汽车、钢铁、粮油、农产品等大型专业物流园区和均川大型综合物流园区建设。

大力优化金融服务环境。健全金融组织体系，突出抓好金融招商，鼓励金融机构创新信贷产品，积极发展保险市场。发挥财政性资源配置和使用效益，激励银行增加信贷规模。扩大市级担保公司业务规模，规范发展小额贷款公司，力争今年社会融资总量、新增贷款分别增长25%和20%以上，其中当年新增贷款65亿元以上，余额存贷比提高2-3个百分点。支持发行15亿元二期城投债券，充分发挥城市投融资平台作用。重视资本市场建设，加大泰晶科技、程力专汽等企业上市培育推进力度，尽快实现企业上市零突破。鼓励企业积极运用中期票据、短期融资券、私募债等新型融资工具，拓宽融资渠道，扩大直接融资比例。进一步完善农村金融服务体系。

大力推进信息化建设与应用。加快信息化与工业化深度融合，鼓励工业企业充分利用现代信息技术，引导发展电子信息、软件服务和物联网等产业，提升通信服务水平和效益。做好湖北“智慧农村”平台试点工作，用信息技术装备现代农业。推进电子政务，提高资源共享和信息化水平，加快建设“智慧随州”。

大力加强人力资源开发。坚持引进与培育相结合，依托博士后产业基地、职业教育、创业岗位等资源和平台，加快壮大专业技术人才和企业管理人才队伍，引导壮大创业者和技术工人群体。培育壮大企业家队伍，真心实意关怀企业家，激发和保护企业家精神。

（七）着力突出项目和投资两大支撑，增强发展后劲。把项目投资作为经济建设主战场，调动一切积极性，优化资源配置，强化要素保障，强力推动项目落地实施、达产见效。

加大项目谋划力度。着眼长远发展，紧盯国家产业导向，充实完善重大项目库，重点围绕产业链条延伸、基础设施完善、生态环境优化、改善保障民生，策划5亿元以上重大项目100个，努力形成储备一批、开工一批、建成一批的梯次推进格局。

突出抓好重点项目。继续开展“项目建设大行动”，完善领导包保、分级负责、检查通报、考核问责等工作机制，坚持每月召开一次项目建设督办会，每季度组织一次现场观摩活动，各县市区和市开发区每季度举行一次5个以上投资过亿元项目的集中开工，力争今年新开工亿元以上项目100个、竣工投产亿元以上项目100个。加强169个市级重点项目的路线图管控，明确责任主体，强化协调服务，突出抓好续建项目建设，力争尽快形成产能。重点抓好工业投资及交通水利、能源电力等基础设施项目建设，积极推进中航奥龙、玉柴专汽产业园、绿能科技等项目早日开工，加快建设。加快推进麻竹高速、宁西铁路增建二线并电气化改造、城际高铁、随县通用机场、京港澳高速广水连接线建设，改造提升107国道广水段。加快实施骨干水利项目，积极促进鄂北水资源配置工程建设。加快风电、生物质发电项目建设，积极谋划太阳能发电项目，全力争取“路口”电站纳入国家建设规划。

扎实开展招商引资。进一步完善招商政策，建立健全目标分解、风险防范、甄别筛选、绩效考评等工作机制，注重引进项目质量和投入产出的性价比，提高招商引资的针对性和专业化水平。创新招商引资方式，继续推行专班招商，重点突出产业链招商、区域招商，促进产业集聚化发展。精心组织招商活动，深化与央企省企的对接合作，积极引导本土企业对外重组。力争今年引进投资过亿元项目40个以上，实际利用外资8000万美元以上。扎实抓好争资立项，力争对上争取各类专款和无偿资金增长10%以上。

（八）着力发展生态文明，建设美丽家园。把生态文明建设提升到更高的战略层面，积极推进“两型”社会建设，努力让随州的天更蓝、地更绿、水更清、城更美、空气更新鲜、家园更美丽。

大力实施城乡三年绿化攻坚计划。广泛开展城镇园林式单位和绿色社区创建活动，全方位实施立体绿化、垂直绿化和房顶绿化。坚持三年封山育林不动摇，力争三年消灭宜林荒山，今年再绿化造林20万亩，推动林下经济发展。加强林业执法监督，严厉打击乱砍乱伐及违规占用林地等行为。

切实抓好国土管理、水资源管理和环境保护工作。优化国土空间利用，实施最严格的耕地保护制度，继续做好城乡建设用地增减挂钩和低丘缓坡地综合开发利用省级试点工作，积极争取用地指标。注重投资强度，实行集约使用，坚决杜绝土地闲置和违法用地。实施最严格的水资源保护制度，加强供水侧和需水侧管理，强化水量水质管理，科学规划利用地表和地下两种水资源。严格执行环境影响评价和“三同时”制度，加大污染防治力度，加强节能减排，确保完成各项约束性指标，深入推进节水、节地、节材型城市建设。

全面开展“洁美家园大行动”。深入推进“三城同创”，对照各项指标体系，高水平、高标准、高质量完成创建任务，力争创建国家园林城市、森林城市和省级环保模范城市全部夺牌。扎实开展“万名干部进万村洁万家”活动，以点带面，示范引导，深化生态村（镇）创建活动，全力打造洁美乡村，建成一批农村新社区。

（九）着力加强社会管理创新和服务，增进民生福祉。始终把保障和改善民生作为最大的政治，加大感情投入、政策投入和资金投入，切实念好衣食住行、医教保业“八字经”。

强化就业和社会保障工作。扶持创业带动就业，拓宽就业渠道，着力解决结构性就业矛盾，突出抓好以高校毕业生为重点的青年就业工作，保持就业形势基本稳定。加强和谐劳动关系机制建设，依法维护职工权益。进一步扩大社会保险参保范围，推进“五险合一”扩面征缴，健全企业退休人员养老金正常增长机制，适时调整失业保险待遇。加快完善全民医保体系，稳步提高待遇标准和服务水平。鼓励和引导社会力量兴办养老机构，提升养老服务水平。扎实做好城乡困难群众、低保五保对象、流浪乞讨人员的帮扶救济工作。

切实办好人民满意的教育。加大教育投入，优化教育资源配置，严格教育行政和教学管理。推进义务教育均衡发展，着力解决城区部分学校“超级大班”的问题，加快改善农村办学条件。抓好学前教育三年行动计划落实，稳步提高入园率。优化发展高中阶段教育，将教育质量提升放在突出位置。规范发展民办教育，巩固提升我市职教品牌；支持随州职业技术学院合理化债，稳定招生规模，推动高职教育健康发展。

大力发展文化医疗卫生等各项事业。推动文化大发展大繁荣，加快完善文化体育惠民设施，加强文化市场监管，让群众广泛享有基本公共文化体育服务。深化医药卫生体制改革，巩固完善基本药物制度，抓好广水公立医院试点改革工作；提高新农合保障水平，不断完善疾病预防和医疗救治体系；加强医德行风建设，依法规范处置医患纠纷。积极创建食品药品安全先进市。继续稳定低生育水平，加强出生人口性别比综合治理。扎实开展全国第三次经济普查，做好红十字会、援疆、无偿献血和老龄工作，积极发展残疾人事业。

全力维护社会和谐稳定。深入推进社会主义核心价值体系建设，广泛开展精神文明创建活动，着力提高城乡人民文明素质和社会文明程度。健全完善基层社会管理和服务体系，全面推行城市社区网格化和农村村务协理模式。深入开展“三民工程”，努力建设幸福社区，完成38个城市社区办公服务场所的规范化建设。强化妇女儿童权益保护，有计划实施农村妇女乳腺癌、宫颈癌免费筛查和治疗补助工作。积极支持工会、共青团等群团组织参与社会管理和服务。继续深化“平安建设大行动”，加强社会管理综合治理，预防和打击各类违法犯罪活动。高度重视信访维稳工作，健全利益协调、矛盾调处机制，加强应急管理，妥善处置突发事件。落实各类安全生产主体责任，切实抓好监管工作，确保人民群众生命财产安全。

进一步加强统计、国调、审计、物价、司法、档案、保密、地方志、气象、水文、邮政、人民防空和防震减灾等工作。加强科技协作，扩大对台经济、文化交流，全面落实民族宗教和侨务政策。深化国防教育，强化国防意识，推进军地融合发展，积极支持驻随部队、武警部队和民兵预备役建设，深入开展共建“双拥模范城”活动，做好军转干部、退役士兵安置和优抚工作。

三、强化政府自身改革和建设

新的历史方位、新的发展蓝图，对各级政府自身改革和建设提出了新的更高要求。我们将着力建设职能科学、结构优化、廉洁高效、人民满意的服务型政府。

（一）切实加大改革创新力度。进一步创新体制机制，破除阻碍发展的思想观念和制度障碍，加快转变政府职能。深化行政审批制度改革，规范审批权限，优化服务流程，坚决将市级审批事项减至200项以内，落实“一周办结制”，真正使随州进入全省行政审批数量最少、时限最短、收费最低的“三最”城市行列。抓好洪山镇和杨寨镇省级行政管理体制改革试点工作。巩固提升大部门制改革成效，加强行政效能建设，探索推广随县省级政府绩效管理试点经验。推进事业单位分类改革，抓好转制转企等工作。深化财税制度改革，加强财源建设，优化财源结构和支出结构，强化税收征管，增强财政实力。

（二）切实提升依法行政能力。加强法治思维和法治方式的运用，严格规范行政行为。积极开展法治城市创建活动，做好法律援助、司法救助、行政复议和行政应诉工作，不断提高政府工作法治化、规范化、制度化水平。完善科学、民主、依法的决策机制，健全重大事项公众参与、专家论证、风险评估、合法性审查和集体讨论等制度。加强政府层级监督，自觉接受人大及其常委会的法律监督和政协民主监督，切实保障人民群众的知情权、参与权、表达权和监督权。

　　（三）切实优化投资发展环境。坚持“产业第一、企业家老大”，着力建设创新创业文化、“重商”文化，塑造“随商”品牌。强化服务企业意识，尤其是要着力提升项目投资建设环节的行政效能。坚决消除“中梗阻”，坚决纠正企业和群众反映强烈的乱收费、乱罚款、乱摊派及不作为、慢作为等突出问题。全面落实中央和省关于改进工作作风的规定，将"作风整顿大行动"引至更高层次更深范畴更实效果。加强公务员队伍建设，讲政治担当、职责担当、能力担当、资格担当，面向经济建设主战场做到担难担曲、敬业专业。健全决策、执行、监督、考核、奖惩工作落实链条，形成雷厉风行的执行体系，确保政令畅通、行政提速。

（四）切实做到为民务实清廉。始终坚持执政为民、以民为本的核心立场，把人民利益放在第一位，讲实话、干实事，言必信、行必果，诚信务实为民排忧解难，实干兴市推进赶超跨越。坚持反腐倡廉与改革发展的“力度统一论”，加强惩治和预防腐败体系建设，严肃查处各类违法违纪案件。不断完善政府内控制度，加大政务公开力度，使权力在阳光下运行。强化行政监察和审计监督，加强对重点领域、重点部门、关键环节的监管，健全完善招投标综合监管体系，推进公共资源交易平台建设。厉行勤俭节约，严格控制“三公”经费支出，实行跟踪问效，坚决把有限的财力用在推动发展、改善民生的关键环节。

今年市政府将继续办好十件实事：（1）扶持创业带动就业，增发小额担保贷款4000万元，城镇新增就业1.8万人，农村劳动力转移就业2万人。（2）扩大社会保险覆盖面，继续提高待遇水平，启动城镇居民大病医疗保险，确保城镇职工、居民医保政策范围内综合报销比例高于全省平均水平，农民医保报销比例达到75%以上。（3）完成保障性安居工程建设任务，推进非公有制企业建立住房公积金制度、扩面新增1万人。（4）发放特困残疾人生活补贴，免费为500名残疾人配发辅助器具，免费为筛查合格的0岁至6岁贫困残疾儿童实施抢救性康复。（5）新建和改扩建20所幼儿园，发放扶贫资助金5000万元、惠及5万名贫困学生。（6）推进市中心医院新院建设。（7）加强饮用水源地治理、监管和保护，基本完成封江水库供水复线建设，再解决24万农村人口的安全饮水问题，减少农村贫困人口1万人。（8）实施“三民工程”，多渠道筹集中心城区社区民心公益基金，其中每个社区市级每年投入20万元、区级每年10万元，连续三年纳入财政预算，用于扶贫济困。（9）加强治安防控体系建设，完善城区视频监控体系，完善城区“五警合一”机制，每个社区组建一支专业治安巡逻队，推进群防群治工作，大幅降低“两抢一盗”发案率，切实增强人民群众安全感。（10）开展“洁美家园大行动”。在主城区新建和改建5-10个街头小游园，建设30座压缩式垃圾中转站（点）、新建和改造20座公厕；改造农村危房5000户以上，完成“一建三改”工程4000个。

各位代表，中华复兴正当其时，湖北崛起方兴未艾，随州发展风帆正劲！让我们在市委的坚强领导下，在市人大、市政协的监督支持下，众志成城，抢抓机遇，实干苦干，提神竞进，为开创“圣地车都”建设新局面、谱写人民美好生活新篇章、成就古老随州新梦想而努力奋斗！
